# The role of microorganisms in the conversion of color class aroma precursors to aroma during air-curing of cigar tobacco leaves

**DOI:** 10.3389/fmicb.2026.1823617

**Published:** 2026-07-14

**Authors:** Songchao Zhao, Yuanyuan Li, Hongyang Si, Zhishun Chai, Yifan Li, Yunkang Lei, Junchun Gao, Mingqin Zhao, Fang Liu

**Affiliations:** 1 College of Tobacco Science, Flavors and Fragrance Engineering & Technology Research Center of Henan Province, Henan Agricultural University, Zhengzhou, China; 2 Staff Development Institute of China National Tobacco Corporation, Zhengzhou, China; 3 Cigar Fermentation Technology Key Laboratory of Tobacco Industry, Chengdu, China; 4 Danzhou City Branch of Hainan Province Tobacco Company, Danzhou, China; 5 Deyang City Branch of Sichuan Province Tobacco Company, Deyang, China; 6 Hongyun Honghe Tobacco (Group) Co., Ltd., Kunming, China

**Keywords:** air-curing, aroma components, cigar tobacco leaf, color class aroma precursors, key functional microorganisms, verification test

## Abstract

**Introduction:**

Air-curing is a key process shaping cigar flavor quality. The mechanism underlying microbial-mediated color-class aroma precursor conversion remains unclear. This study explores their dynamic correlations during air-curing.

**Methods:**

Water content, color class aroma precursors (CCAP, e.g., chlorophyll a, chlorophyll b, β-carotene, etc.) and CCAP-derived aroma components in cigar tobacco leaves were determined during air-curing (AC). To explore the microbial drivers of these dynamic changes, high-throughput sequencing was used to analyze microbial communities across different AC stages. Furthermore, β-carotene-degrading microorganisms were isolated from the leaves using β-carotene-containing solid medium (β-CSM), and their degradation capacity was verified.

**Results:**

Most CCAP contents presented an overall downward trend, while amino acid levels increased initially before declining, and aroma components also changed. Moreover, the structures of bacterial and fungal communities varied in each key phase during the AC process. Partial least squares discriminant analysis identified four core functional microorganisms. *Unclassified*_*f*__*Burkholderiaceae* was closely related to amino acid metabolism of CCAP, whereas *Paenibacillus, Bacillus* and *Alternaria* correlated negatively with β-carotene but positively with its degradation products, indicating roles in β-carotene breakdown and CCAP-to-aroma conversion. Further functional screening and validation confirmed that the screened *Paenibacillus* and *Bacillus* strains possessed prominent β-carotene degradation capacity. In particular, the isolated Paenibacillus strain could degrade β-carotene alone and produce typical cigar aroma components, such as linalool and tabanone 2.

**Discussion:**

The future utilization of microorganisms for the targeted processing of cigar tobacco during production holds significant potential. These findings provide a strong theoretical and practical foundation for the development of high-quality raw materials for manufacturing cigars.

## 1 Introduction

As a high-end tobacco product, cigars have experienced continuous growth in global markets and boast enormous industrial development potential (Custom Market Insights, 2025; Liu et al., 2026). During cigar production, curing process constitutes a crucial step influencing characteristic flavor development and final product quality, which contributes to the distinct flavor characteristics that distinguish cigars from flue-cured tobacco (Zhang et al., 2025). Air-curing (AC) involves drying freshly harvested cigar tobacco leaves under natural or controlled temperature and humidity conditions, leading to a gradual reduction in moisture and desired changes in composition (Zhao et al., 2022). During air curing, leaf color shifts from green to yellow and finally to brown, which is the most pronounced reflection. Substances affecting color changes in tobacco are CCAP (including chlorophyll, carotenoid, browning reaction substance) (Zhang et al., 2018). Degradation of CCAP produces various aroma substances, and the aroma substances influence tobacco quality significantly (Song et al., 2010). As a key component of CCAP, carotenoids have been proven to be transformed into aroma substances with the participation of microorganisms during tobacco fermentation (Wu et al., 2023; Zheng et al., 2022). Analogously, cigar tobacco leaves harbor abundant and diverse microbial communities throughout the entire AC period, whose dynamic metabolic activities actively participate in a series of chemical transformations of tobacco leaf substances (Li et al., 2024; Zhang et al., 2023a). Many microorganisms have been verified to degrade carotenoids and produce aroma compounds, including *Bacillus, Paenibacillus, Pseudomonas, Enterobacter*, and *Sphingobacterium*, etc. mainly by secreting carotenoid cleavage dioxygenases and related enzymes (Rodríguez-Bustamante and Sánchez, 2007; Wu et al., 2023).

Nevertheless, current studies mainly focus on overall changes of tobacco aroma components induced by microorganisms during AC, with limited research on the investigation of metabolic patterns of color-related aroma precursors, along with the mechanisms of CCAP conversion into aromatic substances mediated by microbial communities (Zhang et al., 2025). Revealing how microbial communities regulate the conversion of these CCAP during AC constitutes an essential research orientation to improve raw cigar leaf quality, which also lays a foundation for the optimization of tobacco processing technologies.

## 2 Materials and methods

### 2.1 Materials

The study was conducted at the cigar tobacco production base in Fengcheng Town, Xuanhan County, Dazhou City, Sichuan Province (107°97′E, 31°51′N). The experimental site had a sandy loam soil texture with a pH of 5.6. The soil contained 92 mg/kg of mineralized nitrogen, 8.8 mg/kg of fast-acting phosphorus, and 114.0 mg/kg of fast-acting potassium. The experimental variety was Chuanxue No. 2.

### 2.2 Experimental design and sample collection

Cigar tobacco plants (Chuanxue No. 2) were transplanted on May 6, 2022, with a row spacing of 110 cm and plant spacing of 40 cm. Fertilizer with an N:P_2_O5:K_2_O ratio of 2:1:4 was applied in the field. Middle leaves (11th−12th leaf positions) were harvested at the technical mature stage post-squaring from plants with uniform growth vigor and leaf maturity. Morphologically, these leaves were yellow-green, with 80% of primary veins fully or nearly whitened and approximately 67% of tertiary veins whitening with emerging mature spots. The harvested leaves were subsequently air-cured in a constant-temperature-humidity incubator (Table 1). Samples were collected between 8:00 and 9:00 a.m. across six sequential air-curing stages: fresh leaf stage (T1, day 1), wilting stage (T2, day 3), yellowing stage (T3, day 6), browning stage (T4, day 14), stem-drying stage (T5, day 21), and final air-curing stage (T6, day 30). On each sampling day, representative leaves with consistent visual features matching the corresponding air-curing stage were selected from the same batch and divided into five subsamples for subsequent analysis. One subsample was used for water content determination; another was wrapped in aluminum foil and stored at −80 °C for microbial community analysis; a third was preserved at −80 °C for amino acid quantification; a fourth was dried and ground to measure chlorophyll a, chlorophyll b, β-carotene, lutein, chlorogenic acid, rutin, scopoletin, reducing sugar, soluble protein and aroma-related compounds; and the final subsample was used for microbial strain screening. All determinations were performed in quadruplicate. Table 1 presents the detailed temperature and humidity settings for each air-curing stage.

**Table 1:** Temperature and humidity conditions at different periods during AC.

Index	Wilting stage	Yellowing stage	Browning stage	Stem drying stage
Humidity (%)	80	75	70	45
Temperature (°C)	22	26	30	35

### 2.3 Measurement indices and methods

#### 2.3.1 Moisture content in tobacco leaves

The main and branch veins of the tobacco leaves were excised, the remaining leaf tissue was weighed, and the moisture content of the tobacco was calculated using the heating and drying method (Nirmaan et al., 2020).

#### 2.3.2 Pigments

In a 50 mL Erlenmeyer flask, 2 g of the sample and 25 mL of extraction solution (90% aqueous acetone) were mixed. Subsequently, the mixture was ultrasonicated for 20 min to form an extract that was filtered through an organic 0.45 μm membrane and loaded into a brown 2 mL vial for high-performance liquid chromatography (HPLC) analysis (Lu et al., 2019) using a Waters 2695 instrument (Waters Co., Milford, MA, USA).

#### 2.3.3 Polyphenols

To measure polyphenol contents, 0.01 g of the leaves sample was weighed and poured into a 50 mL triangular flask. Then, 20 mL of 50% methanol was added, and the sample underwent ultrasonic extraction for 30 min. An appropriate amount of the extract was filtered through a 0.45 μm hydrophilic membrane, and the filtrate was loaded into a 2 mL vial to determine the chlorogenic acid, rutin, and scopoletin contents using HPLC (Yu et al., 2013) using the Waters 2695 instrument (Waters Co.).

#### 2.3.4 Reducing sugar and protein assessment

Determination of reducing sugar and protein contents in tobacco leaves was performed according to industry standards YC/T159-2002 and YC/249-2008.

#### 2.3.5 Amino acid

Amino acid content was measured spectrophotometrically using a kit (Beijing Solarbio Science & Technology Co., Ltd., Beijing, China) in accordance with the manufacturer's instructions.

#### 2.3.6 Aroma substance

We used the steam distillation-dichloromethane solvent extraction method for pre-treatment. First, 20 g of tobacco leaf sample was carefully measured and transferred to a 1000-mL round-bottomed flask. To this, 2.0 g citric acid and 600 mL of distilled water were added. The flask was attached to one end of a simultaneous distillation extraction (Beijing Glass Group Company, Beijing, China) device, and to the other end, a flask containing 40 mL of methylene chloride and 1.0 mL internal standard was connected and heated in a water bath at 60 °C. Simultaneous distillation and extraction were performed when the mixture began to boil. After 2.5 h, the organic phase was collected, dried by adding approximately 10 g of anhydrous sodium sulfate, transferred to a glass seed flask, concentrated in a water bath to a volume of approximately 1 mL, and transferred to a chromatographic flask for analysis using an HP5890 II-5972 gas chromatograph (Agilent Technologies Inc., Palo Alto, CA, USA). The detailed analysis parameters are described by Zhan et al. (2011). Aroma substances in the samples were characterized based on authentic standards and the NIST17 database. The quantification (μg/g) was performed by comparing the peak area with the peak area of the internal standard substance, and the calculation was conducted using the formula shown below. The internal standard was prepared by weighing 0.7 g of phenethyl acetate, followed by addition of 100 mL of anhydrous ethanol. Dilute 40 times when used.


$$\begin{array}{l} \text{Aroma substance }\left(\text{μg}/\text{g}\right)=\text{Peak area of aroma substance}\times \\ \;\frac{\text{content of internal standard}}{\text{peak area of internal standard}} \end{array}$$

### 2.4 DNA extraction and sequencing

We extracted DNA according to a previously described protocol (Ye et al., 2017). Genomic DNA was extracted from cigar tobacco samples using the E.Z.N.A.^®^ Soil DNA Kit (Omega Bio-tek, USA) according to the manufacturer's instructions. Next, the quality of the extracted DNA was evaluated via 1% agarose gel electrophoresis. DNA concentration and purity were determined using a NanoDrop 2000 instrument (Thermo Fisher Scientific Inc., Waltham, MA, USA). For bacterial community profiling, the V5–V7 hypervariable region of the 16S rRNA gene was amplified with primers 799F 5′-AACMGGATTAGATACCCKG3′) and 1193R 5′-ACGTCATCCCCACCTTCC3′); this primer pair was selected to minimize co-amplification of plant plastid (chloroplast and mitochondrial) 16S rRNA sequences, which is critical for tobacco leaf matrices, as chloroplast sequences typically dominate amplicon libraries when conventional V3–V4 primers are used. This combination delivers robust taxonomic resolution and effectively reduces interference from host plant-derived sequences in tobacco leaf microbiome analysis. The fungal ITS region was amplified via PCR as described by Liu et al. (2021). All PCR amplifications were performed in triplicate per sample. The PCR conditions included initial denaturation (95 °C, 3 min), 35 cycles of denaturation (95 °C, 30 s), annealing (55 °C, 30 s), extension (72 °C, 45 s), and final extension (72 °C, 10 min). Triplicate PCR products from the same sample were pooled, verified by 2% agarose gel electrophoresis, and purified using the AxyPrep DNA Gel Extraction Kit (Axygen Biosciences Inc., Union City, CA, USA) following the manufacturer's instructions. Purified amplicons were quantified with the Quantus™ Fluorometer (Promega Co., Madison, WI, USA), and sequencing libraries were constructed using the NEXTFLEX™ Rapid DNA-Seq Kit (Bioo Scientific Co., Austin, TX, USA). Sequencing was performed on the Illumina MiSeq PE300 platform by Shanghai Majorbio Co., Ltd. (Shanghai, China).

### 2.5 Processing of sequencing data

First, fastp version 0.20.0 (Chen et al., 2018) (https://github.com/OpenGene/fastp) and FLASH version 1.2.7 (Magoč and Salzberg, 2011) (https://ccb.jhu.edu/software/FLASH/) were used for quality control and raw sequence assembly, respectively. Next, the DADA2 plug-in (Callahan et al., 2016) of the QIIME2 platform (Bolyen et al., 2019) was used for noise reduction in the sequences that had been optimized through quality control and assembly using default parameters, yielding amplicon sequence variants (ASVs). The naive Bayes classifier in QIIME2 was used for the taxonomic analysis of ASVs based on the SILVA 16S rRNA database (http://www.arb-silva.de/, version 138) and the UNITE 8.0 ITS database (https://mothur.org/wiki/unite_its_database/, version 8.99).

### 2.6 Medium and reagent preparation

Luria-Bertani (LB) medium was employed for bacterial culture (Lee and Gilmore, 2005). Referring to the method described by Zorn et al. (2003), β-carotene-containing medium (β-CM) was prepared as follows. 1.0 g/L KH_2_PO_4_, 0.5 g/L MgSO_4_·7H_2_O, 3.0 g/L NaNO_3_, 0.01 g/L FeSO_4_·7H_2_O, 0.5 g/L KCl, 30.0 g/L sucrose, and 2.0 g/L yeast extract were combined and sterilized at 115 °C for 20 min. Subsequently, a stock solutionβ-carotene of 20% was introduced to the medium after sterilization to detect β-carotene-degrading microorganisms. Solid medium was added with 1.5% agar powder.

### 2.7 Microbial screening and aroma collection

First, 2 g samples of tobacco leaves were placed into 100 mL of sterile normal saline. The mixture was then incubated at 30 °C and shaken at 160 rpm for 8 h. Next, 50 μL of the supernatant was collected, inoculated on β-carotene-containing solid medium (β-CSM), and incubated for 24 h at 30 °C for bacteria and 25 °C for fungi. After incubation, colonies that discolored the medium were selected, streaked, and separated until single colonies were isolated. Depending on the strain, isolated single colonies were inoculated into LB liquid medium and incubated for 24 h at 30 °C, while shaking at 160 rpm. Subsequently, the strains were inoculated into 30 mL of β-CM at an inoculum concentration of 10% and incubated for 24 h at 30 °C, while shaking at 160 rpm. The color change of the liquid medium was observed afterwards. These steps were performed under sterile conditions.

After the microorganisms were cultured in the β-CM for 24 h, the medium was centrifuged at 4000 r/min. The supernatant was collected in a separatory funnel (Zhengzhou Zhongtian Experimental Instrument Co., Ltd., Zhengzhou, China), dichloromethane was added (dichloromethane volume: volume of supernatant = 1:1), and shaken up and down for 1 min, after which it was left to stand for 20 min for the extraction of aroma components. Afterwards, it was concentrated and tested according to section 2.3.6. The control treatment involved inoculation of bacteria in a β-CM supplemented with a β-carotene-free stock solution for cultivation.

### 2.8 β-carotene degradation efficiency

To create a standard curve for β-carotene, 0, 20, 40, 60, 80, 100, 120, and 140 μL aliquots of β-carotene stock solution were added to distilled water to a final volume of 3.25 mL, and the absorbance was measured at 450 nm using distilled water as a blank. Then, the data were plotted graphically to determine the linear relationship between the concentration of β-carotene solution and absorbance, described by y = 23.801x – 0.0553 (*R*^2^ = 0.9943), where y is the concentration of β-carotene in aqueous solution (μmol/L), and x is the absorbance of the aqueous solution of β-carotene.

To assess β-carotene degradation efficiency, β-carotene-degrading microbial strains obtained by β-CM (Section 2.6) were inoculated in the screening medium and incubated for 24 h. The degradation efficiency of β-carotene was then determined based on absorbance values and standard curves (Long et al., 2022).

### 2.9 Microbial identification

The target strains were screened as described in Section 2.7 and the strains were identified based on the screening results. The selected strains were sequenced and classified using 16S rRNA gene sequencing with the primers 27F/1492R.

### 2.10 Data processing

Physical and chemical data from the cigar tobacco AC process were analyzed using Microsoft Excel 2016 (Microsoft Co., Redmond, WA, USA). SPSS version 21.0 (IBM Corp., Armonk, NY, USA) was used to conduct a one-way analysis of variance to determine statistically significant differences (*P* < 0.05) between data groups. The Majorbio Cloud Platform (with built-in QIIME2 analysis pipeline, cloud.majorbio.com) was used to analyze microbial community composition, and one-way ANOVA and Tukey-Kramer test were applied to analyze alpha diversity indices. Constrained principal coordinate analysis (CPCoA) based on Bray-Curtis distances, as well as correlation analysis using Pearson's calculation method, were performed using the Lingbo MicroClass platform (http://www.biomicroclass.com/). The CCAP and aroma components were visualized and analyzed using the Biomarker cloud tool (http://www.biomarker.com.cn), and each row of data was clustered and normalized. Samples were analyzed by principal component analysis (PCA), hierarchical cluster analysis (HCA), and PLS-DA, and variable importance in projection (VIP) values were calculated using SIMCA 14.1 (Umetrics, Umea, Sweden). SPSS version 21.0 (IBM Corp., Armonk, NY, USA) was used for correlation analysis, and Photoshop (Adobe Systems Incorporated, San Jose, CA, USA) was applied to visualize the correlation results. MEGA 5 (Tamura et al., 2011) was used to construct a microbial community phylogenetic tree.

## 3 Results and discussion

### 3.1 Changes in water, CCAP, and aroma components in cigar tobacco during AC

**Figure 1:**
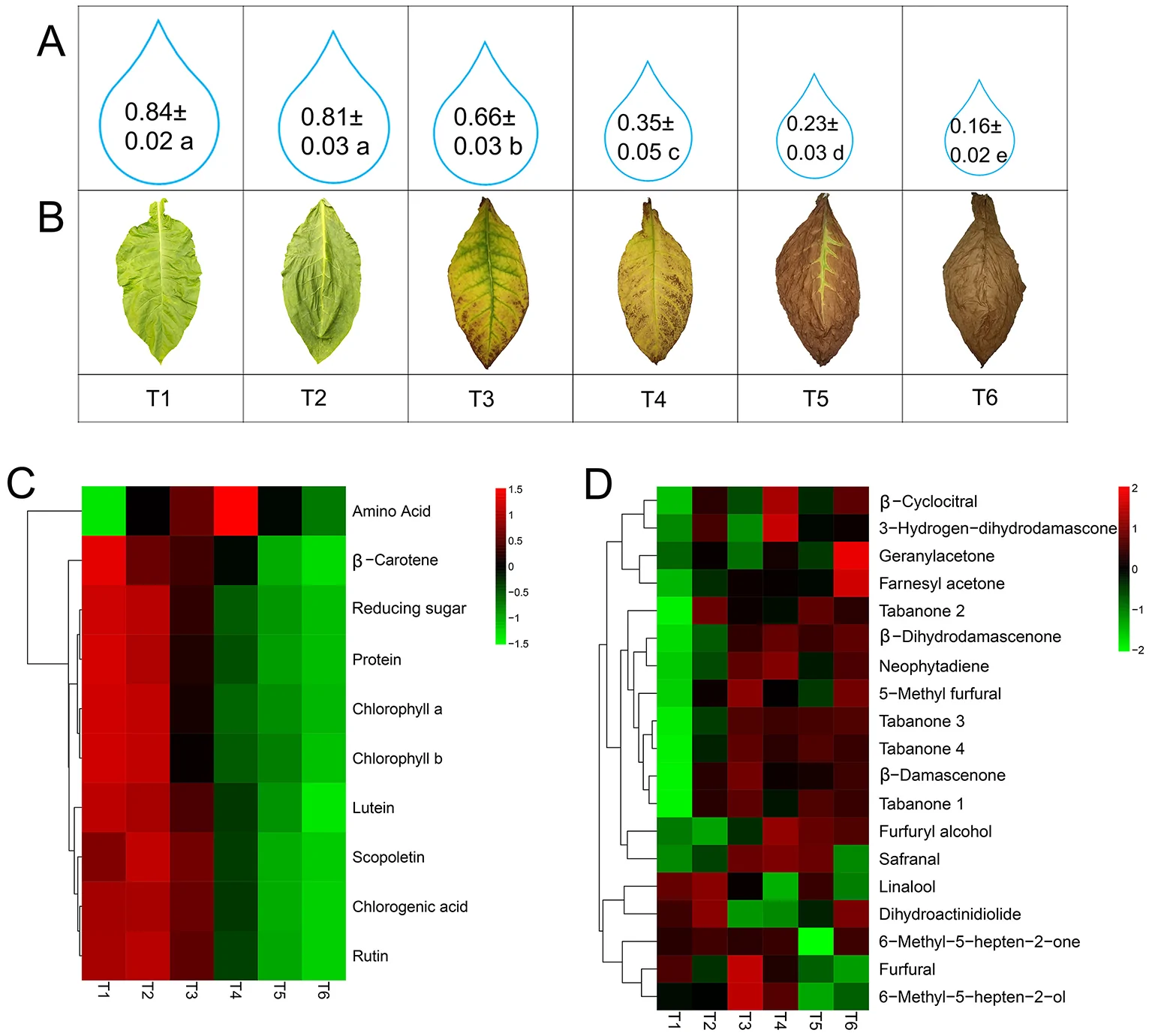
Changes in cigar tobacco leaves during AC. **(A)** leaves moisture content; **(B)** leaves color; **(C)** heatmap analysis of CCAP; **(D)** heatmap analysis of aroma composition. Legends for **(C)** and **(D)** show the log-transformed scores of their contents. From green to red represents the content from low to high. T1, fresh tobacco leaves; T2, wilting; T3, yellowing; T4, browning; T5, stem drying; T6, end of AC. Values are presented as mean ± standard deviation. A significant difference is denoted by distinct letters (*P* < 0.05).

Unlike the field environment, there is no water supply during the AC process; thus, cigar tobacco leaves dry naturally, exhibiting water loss during AC (Figure 1A). The appearance of the cigar leaves changed substantially during the AC process (Figure 1B), consistent with the changes in CCAP contents (Supplementary Table S1 and Figure 1C) (Wang et al., 2022). Further, the aroma components produced by CCAP changed during AC (Supplementary Table S2 and Figure 1D). Chlorophyll a and chlorophyll b, the green pigments in tobacco leaves, undergo degradation during AC, producing the aromatic compound neophytadiene (Wahlberg et al., 1977). Similarly, carotenoids (yellow pigments, mainly β-carotene and lutein) are broken down into terpenoids and other aromatic substances (Karppinen et al., 2016; Xiao et al., 2022). Reducing sugars (browning reaction substances) react with amino acids and proteins (browning reaction substances) in a Maillard reaction, impacting tobacco leaf quality significantly by promoting the characteristic aroma and brown color of cigar tobacco leaves (Van Boekel, 2006). Polyphenols (browning reaction substances) are also aroma substances in their own right, and undergo moderate oxidation during AC to produce quinones, and the carbonyl groups in the quinones are involved in the Maillard reaction, again browning the tobacco and increasing its aroma (Bork et al., 2024; Lu et al., 2019). During the AC process, a pronounced decrease in chlorophyll content was observed compared with that of other constituents. Chlorophyll a and chlorophyll b decreased by 94.66% and 96.46%, respectively. Amino acids in the browning reaction substances showed a tendency to increase and then decrease, which is due to the degradation of proteins to produce amino acids, causing the accumulation of amino acids, and then a decrease (Lu et al., 2020). In addition, furfural showed a tendency to decrease during the decrease in amino acids, which may be due to the Maillard reaction between the amino group in amino acids and the carbonyl group in furfural.

### 3.2 Combined analysis of CCAP and aroma components

**Figure 2:**
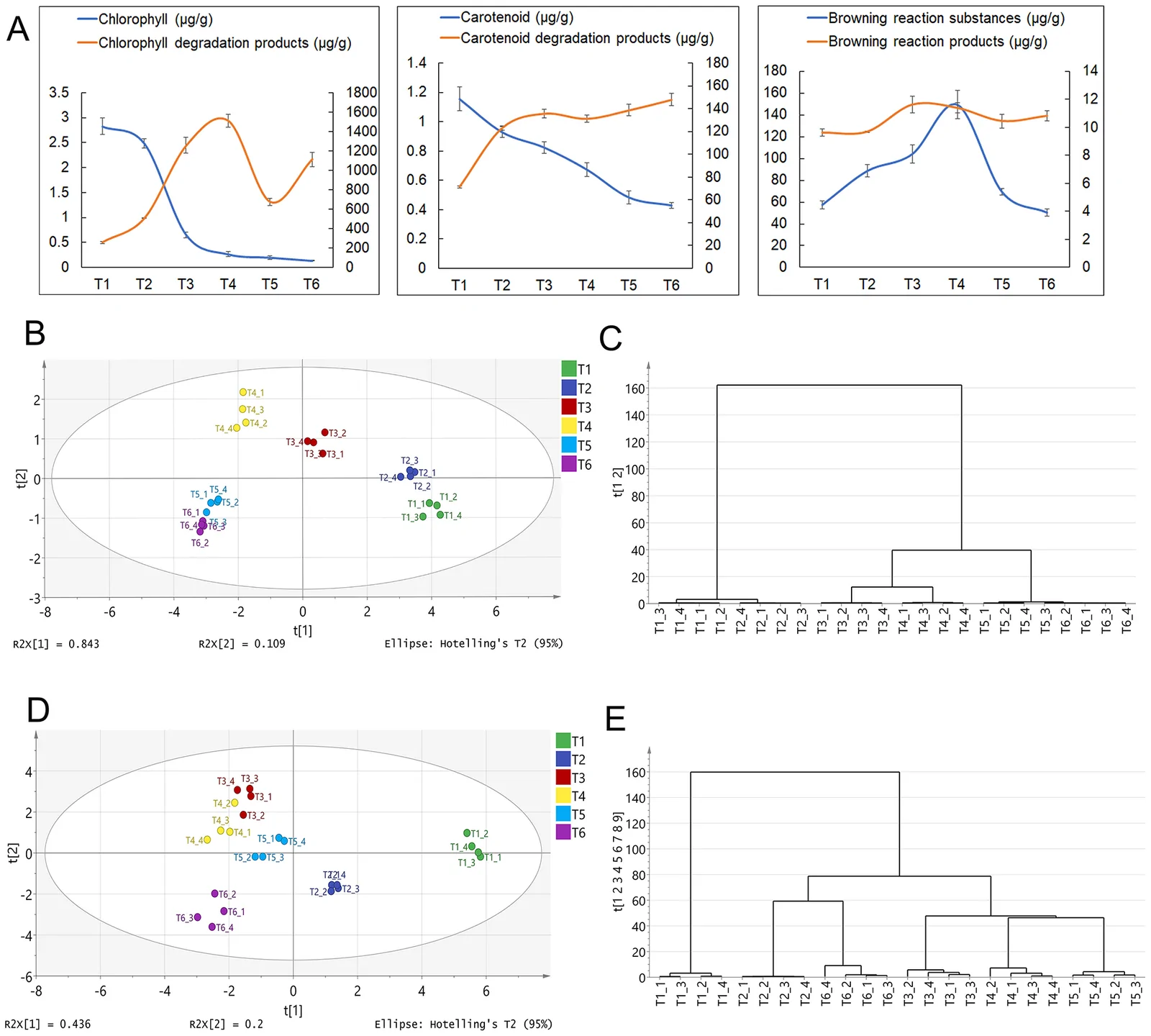
Comprehensive analysis of CCAP and aroma components. The blue line represents the CCAP category and the orange line represents the aroma component category **(A)**. PCA analysis **(B)** and HCA analysis **(C)** of CCAP, and PCA analysis **(D)** and HCA analysis **(E)** of aroma constituents of cigar tobacco during the AC process. Error bars represent mean ± standard deviation. T1, fresh tobacco leaves; T2, wilting; T3, yellowing; T4, browning; T5, stem drying; T6, end of AC.

We performed a combined analysis of the changes in the categories of CCAP during AC and the changes in the categories of aroma components during AC (Figure 2A). Aroma substances in tobacco showed a tendency to increase, then decrease, and then increase during AC. In the early stage of AC, the high water content in the tobacco leaf directly affected the intensity of cellular metabolism, resulting in the degradation of CCAP and the production of aroma components (Song et al., 2010). At mid-to-late discoloration (T3–T5), aroma declined partly due to membrane damage increasing volatile escape (Zhao et al., 2019); neophytadiene dropped sharply at T4–T5 as chlorophyll degradation ceased, coupled with enzymatic degradation and volatilization (Zhang et al., 2023a). By late AC (T5–T6), the contents of aroma components increase moderately. Meanwhile, the total microbial communities may retain the potential to degrade CCAP precursors and generate aroma substances through the aforementioned reactions, which could act as a vital contributor to the aroma recovery during this stage (Huang et al., 2022; Wang et al., 2021). PCA and HCA indicated that AC altered CCAP (Figures 2B, C) and aroma compounds (Figures 2D, E) in cigar tobacco leaves. CCAP samples clustered regularly by curing time with consistent PCA and HCA results. For aroma components, the first two PCs only explained 63.6% total variance and samples failed to cluster chronologically. It is speculated that aroma formation is complex and may be jointly regulated by endogenous enzymes, Maillard reaction, and microbial metabolism beyond curing duration (Zhang et al., 2023a).

### 3.3 Analysis of microbial community structure in cigar tobacco during AC

A total of 1,200,486 bacterial sequencing reads and 2,704,932 fungal sequencing reads were obtained from all cigar tobacco samples using QIIME2 (2020.2 release). To account for potential bias from uneven sequencing depth between samples, all sequencing counts for each sample were normalized to the sample with the lowest sequencing depth. Additionally, chloroplast and mitochondrial sequences were eliminated. For subsequent analyses, 38,733 valid bacterial sequences and 88,409 valid fungal sequences per sample were used. From these, a total of 9,906 bacterial ASVs and 1,372 fungal ASVs were identified.

**Figure 3:**
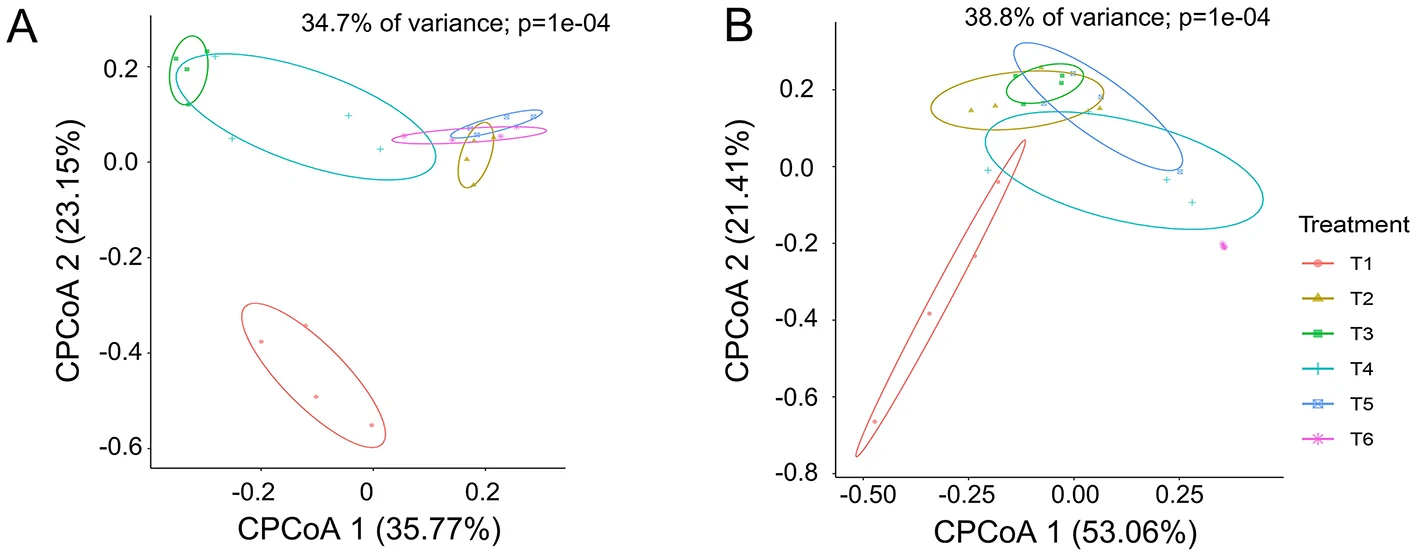
Comparison of bacterial **(A)** and fungal communities **(B)** at different phases. Constrained principal coordinate analysis (CPCoA) of bacterial **(A)** and fungal **(B)** communities at different phases based on Bray-Curtis distances. T1, fresh tobacco leaves; T2, wilting; T3, yellowing; T4, browning; T5, stem drying; T6, end of AC.

To visualize the effect of the AC process on the microbial communities in cigar tobacco, differences in ASV levels of microbial communities at different phases of the AC process were compared using CPCoA (Figures 3A, B). CPCoA revealed pronounced clustering of both bacteria (Figure 3A; 34.70% of the variance, *P* = 1 × 10^−4^) and fungi (Figure 3B; 38.80% of the variance, *P* = 1 × 10^−4^) in samples collected at different AC stages in the cigar tobacco leaves. The abundance (ACE; Supplementary Figure S1A) and diversity (Shannon; Supplementary Figure S1B) indices of bacteria exhibited an M-shaped pattern during the AC process. Both parameters peaked at T2 and T5, with significantly higher values than those at T1, T3, and T4 (*P* < 0.05). The ACE and shannon index of fungi (Supplementary Figure S1C, D) exhibited an M-shaped structure similar to that of bacteria. In the present study, the changes in bacterial communities were more similar to the changes in aroma composition, which may be due to the greater importance of bacteria in the formation of tobacco aroma (Zheng et al., 2022). The results demonstrate that the various stages of AC influenced the bacterial and fungal populations considerably.

### 3.4 Analysis of microbial alpha diversity and community structure in cigar tobacco during AC

**Figure 4:**
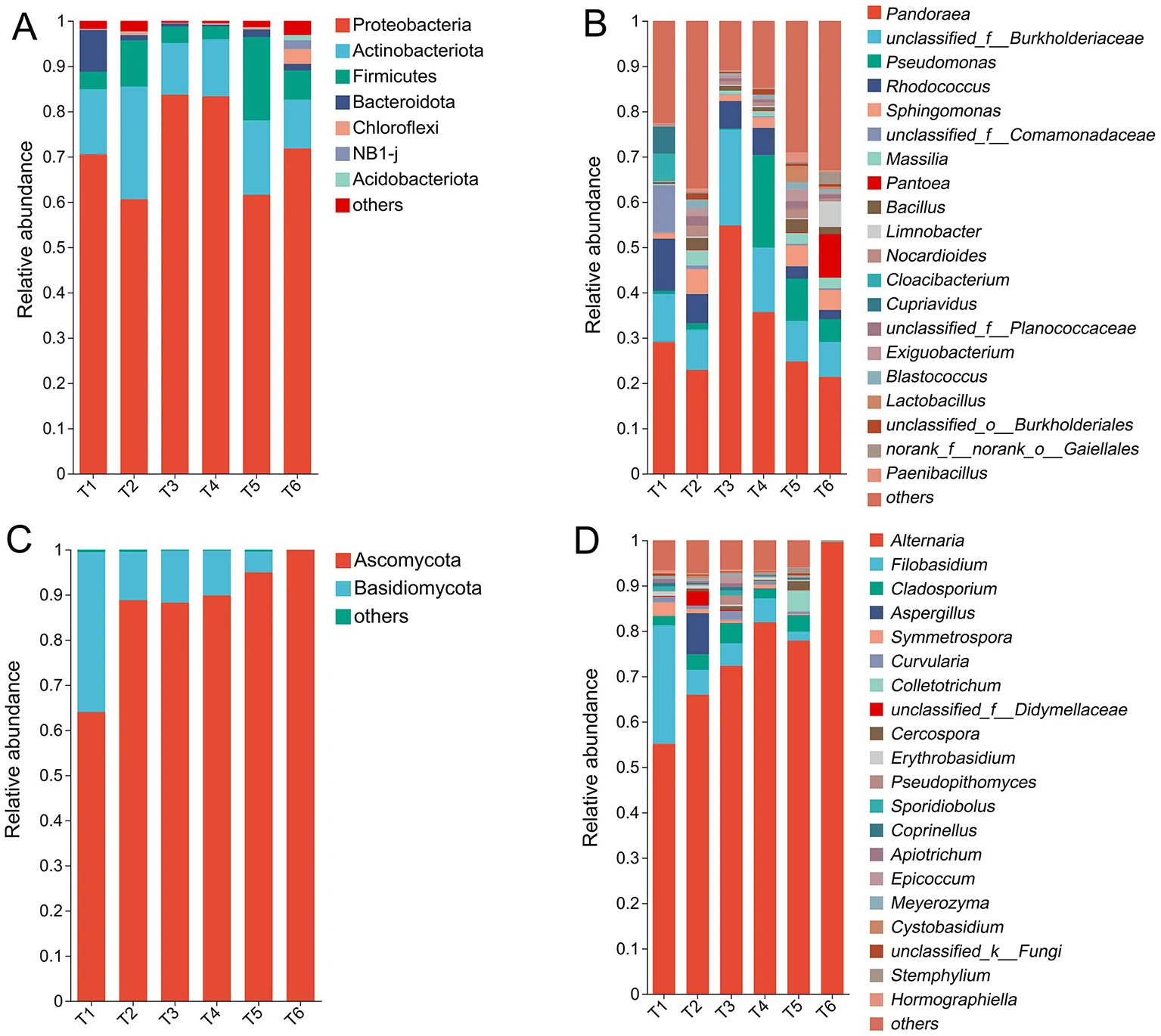
Community structure of tobacco microorganisms. Relative abundance of bacterial **(A, B)** and fungal **(C, D)** communities at the phylum and genus levels. T1, fresh tobacco leaves; T2, wilting; T3, yellowing; T4, browning; T5, stem drying; T6, end of AC.

During the AC process, seven major bacterial phyla were identified (Figure 4A), namely Proteobacteria, Actinobacteriota, Firmicutes, Bacteroidota, Chloroflexi, NB1-j, and Acidobacteriota, which accounted for 96.90%−99.57% of all bacteria. Other phyla were also detected; however, their abundances were low (<1%). At the genus level (Figure 4B), all treatments showed a high abundance of *Pandoraea* spp.; the abundance at T3 was significantly greater than those at other treatment stages (*P* < 0.05). Basidiomycota and Ascomycota comprised the majority of fungal phyla (Figure 4C). Ascomycota accounted for a higher abundance at each phase and increased gradually with curing, reaching 99.95% at T6. In contrast, Basidiomycota exhibited decreasing abundance as curing progressed, with the lowest abundance (0.05%) occurring at T6. At the genus level (Figure 4D), *Alternaria* exhibited a high abundance in all curing phases and decreased gradually during the curing process from 56.22% at T1 to 9.63% at T6. The abundance of *Filobasidium* was also high, at T1 (26.65%), but decreased rapidly thereafter. The results indicate that the AC results in changes in microbial communities.

**Figure 5:**
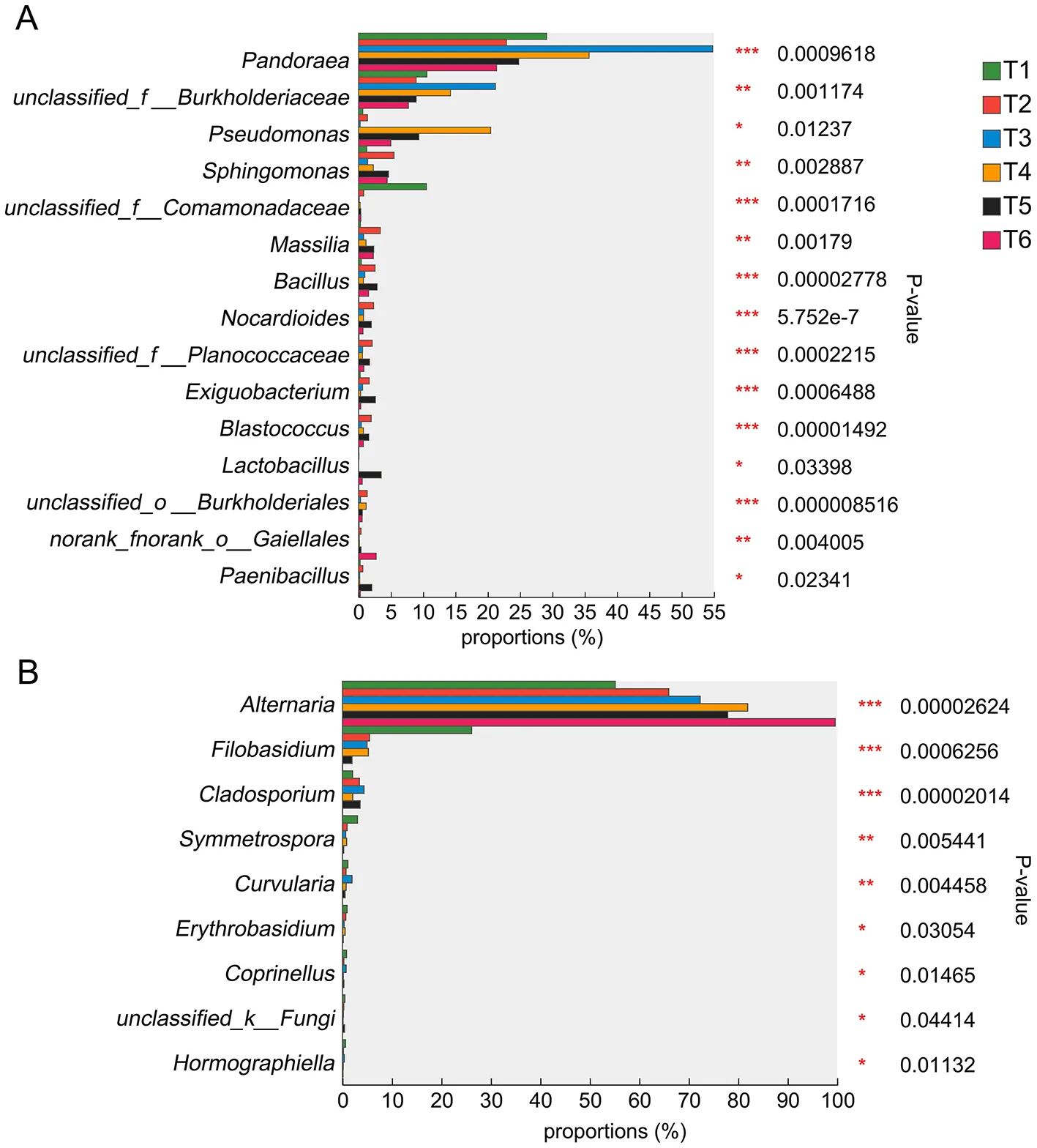
Differences between bacteria **(A)** and fungi **(B)** at the genus level during AC. T1, fresh tobacco leaves; T2, wilting; T3, yellowing; T4, browning; T5, stem drying; T6, end of AC.

Proteobacteria, a bacterial phylum, and Ascomycota, a fungal phylum, exhibit remarkable adaptability, showcasing their ability to efficiently utilize nutrients in low-nutrient environments (Chen et al., 2017; Liang et al., 2021); therefore, they were highly abundant during curing. The bacterial genus *Pseudomonas* and fungal genus *Alternaria* proliferated markedly in the late stage of AC (Figures 5A, B). This phenomenon was likely attributed to the strong environmental adaptability and unique metabolic capacity of *Pseudomonas* (Zhang et al., 2023b). Meanwhile, as a typical saprotrophic fungus, Alternaria could utilize leaf tissues with disrupted cellular structures to obtain nutrients (Zhao et al., 2026). As a predominant fungus during tobacco curing, *Alternaria* has been reported to participate in the degradation of carotenoids (e.g., β-carotene) (Tiwari and Upadhyay, 2016) and the formation of characteristic flavor compounds such as β-ionone (Pan et al., 2019). Thus, it may correlate with pigment degradation and variations in aroma components. *Bacillus* and *Paenibacillus* trends were similar to the aroma trends, which may be attributed to the capacity of both *Bacillus* and *Paenibacillus* to affect substances such as plastid pigments and proteins in tobacco to enhance the aroma of tobacco (Huang et al., 2022; Ye et al., 2019). Other differential genera such as *Pandoraea* and *Pseudomonas* were able to degrade lignin and aromatic compounds to improve the aroma of tobacco (Chen et al., 2008; Liu et al., 2018).

### 3.5 Correlation analysis of key functional microorganisms and environmental factors during cigar tobacco AC

**Figure 6:**
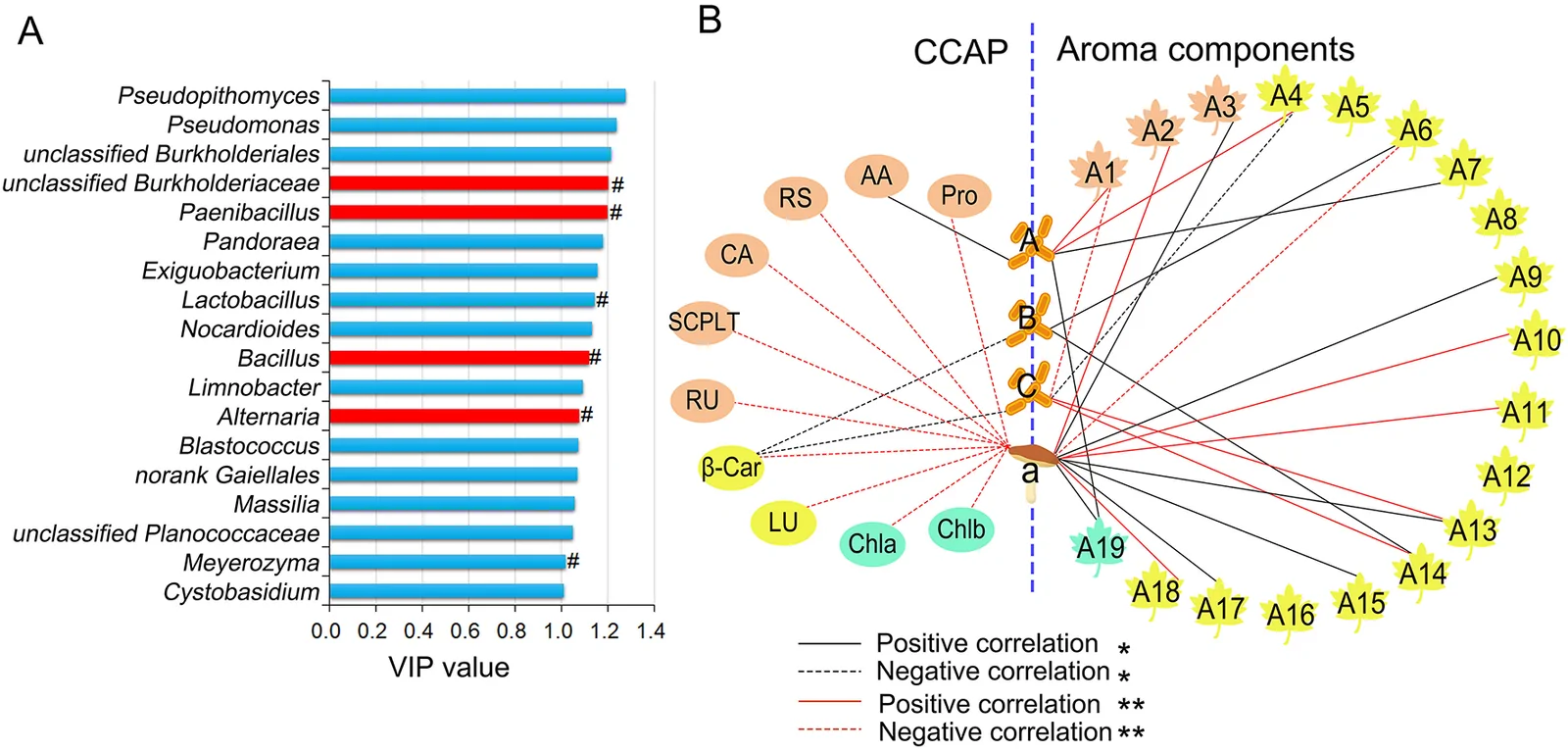
Microbial VIP values based on PLS-DA analysis **(A)** and correlation of key functional microorganisms with environmental factors **(B)**. #: indicates that the microorganism has significant correlations with both CCAP and aroma components (*P* < 0.05). Red bar: microorganism abundance in each phase of AC was > 0.1% under # conditions. (Chla) chlorophyll a. (Chlb) Chlorophyll b. (LU) Lutein. (β-Car) β-Carotene. (CA) Chlorogenic acid. (RU) Rutin. (SCPLT) Scopoletin. (A) unclassified_f_Burkholderiaceae. (B) Paenibacillus. (C) Bacillus. (a) Alternaria. (A1) Furfural. (A2) Furfuryl alcohol. (A3) 5-Methyl furfural. (A4) 6-Methyl-5-hepten-2-ol. (A5) 6-Methyl-5-hepten-2-one. (A6) Linalool. (A7) Safranal. (A8) β-Cyclocitral. (A9) β-Damascenone. (A10) β-Dihydrodamascenone. (A11) Geranylacetone. (A12) Dihydroactinidiolide. (A13) Tabanone 1. (A14) Tabanone 2. (A15) Tabanone 3. (A16) Tabanone 4. (A17) 3-Hydrogen-dihydrodamascone. (A18) Farnesyl acetone. (A19) Neophytadiene.

We used the PLS-DA model to understand the correlativity between microorganisms (top20, independent variables) and CCAP (dependent variables) and aroma components (dependent variables) (Supplementary Figure S2). The results showed that the model fit was good, and there was no overfitting. A total of 18 microorganisms were identified as functional microorganisms, including 14 bacteria and 4 fungi, during the process of aroma formation in AC (Supplementary Table S3). Meanwhile, we screened for microorganisms with significant correlations (*P* < 0.05) with both CCAP and aroma components, as well as microorganisms with relative abundance greater than 0.1% at each AC stage (VIP value >1, Figure 6A). This analysis revealed three genera of bacteria, including *unclassified_f__Burkholderiaceae, Paenibacillus*, and *Bacillus*, and one genera of fungi, namely *Alternaria*. This shows that the production of aroma components in tobacco is mainly associated with bacteria (Zheng et al., 2022).

We visualized the correlation between these four key functional microorganisms and CCAP and aroma components (Figure 6B). From the results, it can be seen that *unclassified_f __Burkholderiaceae* was positively correlated (*P* < 0.05) with amino acids in CCAP. The remaining three genera were negatively correlated with CCAP but were positively correlated with aroma substances (*P* < 0.05). The results indicated that the key functional microorganisms mainly played a role in promoting the conversion of CCAP into aroma components. We found that all three genera were negatively correlated with β-carotene and mainly positively correlated with carotenoid degradation products. *Bacillus* is well known to degrade carotenoids via secreted enzymes and organic acids (Zhang et al., 2025; Ahrazem et al., 2016). Huang et al. (2022) also confirmed that Bacillus spp. were capable of degrading carotenoids to produce aroma substance. *Alternaria* produces carotenoid cleavage dioxygenases and cytochrome P450 monooxygenases to break down β-carotene and lutein into volatile aroma compounds (Zorn et al., 2003; Rodríguez-Bustamante and Sánchez, 2007). Recent studies have verified the ability of *Paenibacillus* to degrade β-carotene. Gong et al. (2023) found that an enzyme preparation from Paenibacillus significantly elevated carotenoid degradation products, implying a functional link between Paenibacillus and carotenoid metabolism. More directly, Sun et al. (2026) demonstrated that *Paenibacillus oceanisediminis* CJ-2 efficiently degrades carotenoids via key enzymes including CRTZ and CCD7, yielding β-damascenone, dihydroactinidiolide and other tobacco aroma components. Accordingly, we hypothesized that *Paenibacillus, Bacillus*, and *Alternaria* collectively degrade β-carotene to produce aroma substances. On this basis, we screened microorganisms with high β-carotene-degrading capacity to verify this hypothesis.

### 3.6 Verification test

**Figure 7:**
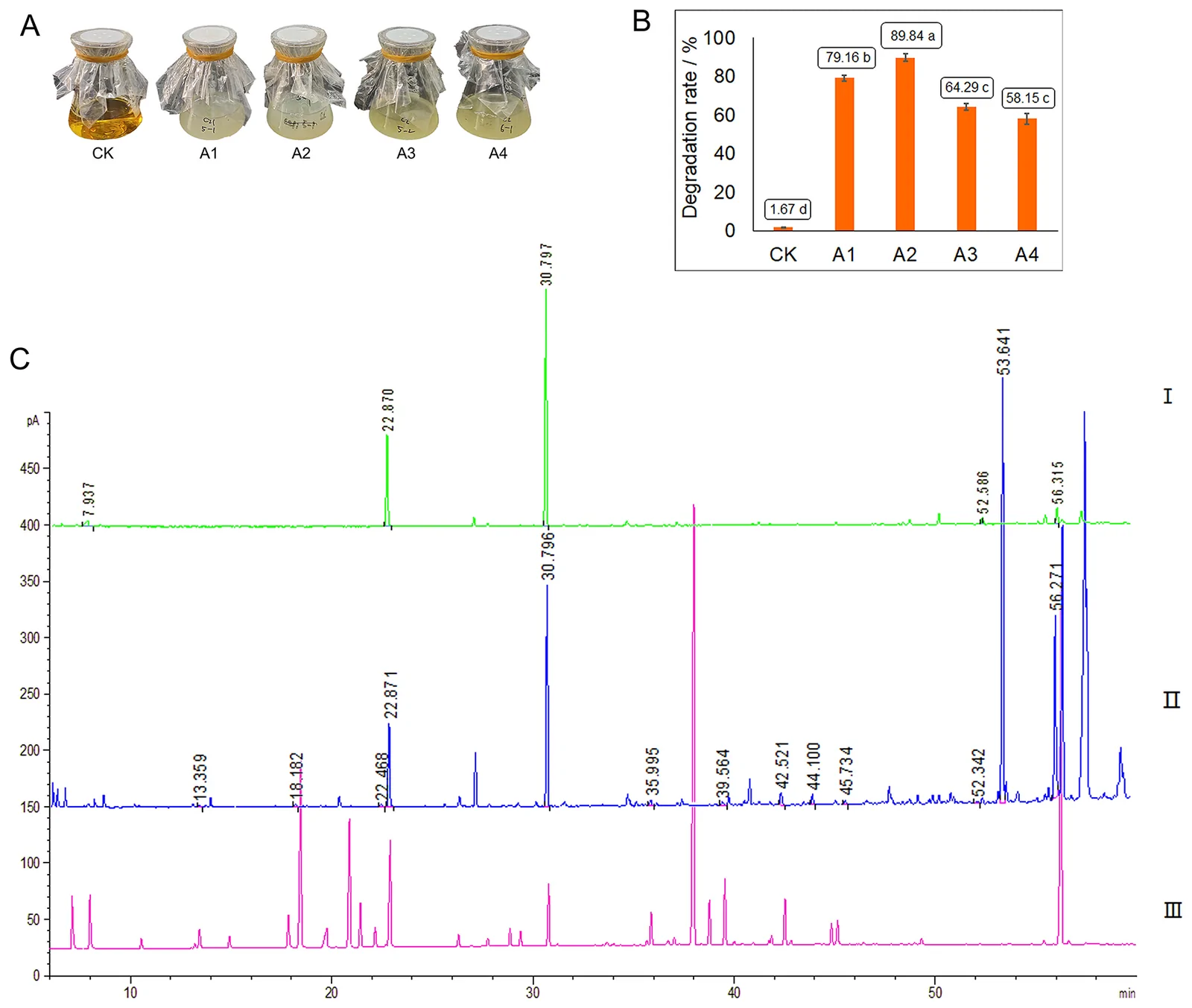
Identification of microorganisms for treatment. **(A)** Degradation of β-carotene. **(B)** Degradation rate of β-carotene by strains. **(C)** Chromatograms of β-carotene degradation products determined by GC-MS, I is the chromatogram of the control group without β-carotene, II is the chromatogram of the treatment group with β-carotene, and III is the chromatogram of the standard. Where X-axis indicates time and Y-axis indicates abundance of compounds.

After screening, four strains of bacteria capable of degrading β-carotene were observed, which were numbered A1, A2, A3, and A4 (Figure 7A). The degradation rate of β-carotene was highest in A2 at 89.84% (Figure 7B). The molecular identification of these four bacterial strains and the construction of their phylogenetic tree (Supplementary Figure S3) showed that A1, A3, and A4 were of the genus *Bacillus* and A2 was of the genus *Paenibacillus*. Among them, A3 and A4 were of the same species, which might be the reason for their similar efficiency of β-carotene degradation. Sequence comparison was performed between the nearly full-length 16S rRNA sequences of the four strains and ASV sequences derived from tobacco leaves. The results revealed that the V5–V7 hypervariable regions of A1, A2, A3 and A4 shared 100% sequence similarity with ASV17, ASV57, ASV501 and ASV501, respectively. All relevant sequences are summarized in Supplementary Table S4 and deposited in the NCBI database. These findings establish a reliable correlation between the functional isolates and core microbial taxa, suggesting that these degradative bacteria are members of tobacco leaf microbiota. We collected the supernatant of *Paenibacillus* cultures for the determination of aroma components to further validate our speculation that *Paenibacillus* degraded β-carotene and produced aroma. The results of the control and treatment groups showed that *Paenibacillus* was able to produce aroma substances. Meanwhile, the degradation of β-carotene by *Paenibacillus* produced substances, such as linalool and tabanone 2 (Table 2 and Figure 7C), and these two middle aroma substances showed a significant positive correlation with *Paenibacillus* during AC. However, other aroma products were not significantly positively correlated with *Paenibacillus* during AC, probably because *Paenibacillus* produces this substance in synergy with other microorganisms (Grady et al., 2016; Rodríguez-Bustamante et al., 2009). This also identifies another function of *Paenibacillus*.

**Table 2:** Comparison of aroma products in different treatments.

Treatment	CAS	Retention time (min)	Aroma substance	Content (μ/g)
A2	100-52-7	13.359	Benzaldehyde	0.056
	62565-79-1	18.182	3,4-Dimethylfuran-2,5-dione	0.146
	78-70-6	22.468	Linalool	0.245
	60-12-8	22.871	Phenethyl alcohol	4.132
	103-45-7	30.796	Phenethyl acetate (internal standard)	9.931
	1937-54-8	35.995	Solanone	0.264
	3796-70-1	39.564	Geranylacetone	0.219
	17092-92-1	42.521	Dihydroactinidiolide	0.749
	13215-88-8	44.10	Tabanone 2	0.669
	13215-88-8	45.734	Tabanone 3	0.224
	54878-25-0	52.342	Solavetivone	0.174
	504-96-1	53.641	Neophytadiene	22.466
	1117-52-8	56.271	Farnesyl acetone	9.893
CK	98-00-0	7.937	Furfuryl alcohol	0.435
	60-12-8	22.870	Phenethyl alcohol	4.247
	103-45-7	30.796	Phenethyl acetate (internal standard)	9.931
	54878-25-0	52.586	Solavetivone	0.051
	1117-52-8	56.315	Farnesyl acetone	0.682

The results verified our speculation that *Paenibacillus* is able to degrade β-carotene and produce aroma components. These results suggest that key functional microorganisms play a major role as degraders in the conversion of CCAP to aroma. Nevertheless, the current conclusion is obtained based on *in vitro* liquid culture tests, and further verification experiments on cigar tobacco leaves require investigation in subsequent studies.

## 4 Conclusion

The microbial community of cigar tobacco leaves exhibited dynamic changes during AC. As the tobacco leaves underwent AC, they gradually dried and lost moisture, leading to the degradation of CCAP and production of aroma substances. Further investigations revealed that *Paenibacillus* spp. and *Bacillus spp*. in tobacco leaves can degrade β-carotene. Among them, *Paenibacillus* spp. was able to degrade β-carotene alone and produce aroma components, such as linalool and tabanone 2. The utilization of microorganisms for the targeted drying of cigar tobacco in future production holds significant potential. The findings of this study establish a theoretical and practical foundation for producing high-quality raw materials for cigar manufacturing.

## Data Availability

The datasets presented in this study can be found in online repositories. The names of the repository/repositories and accession number(s) can be found in the article/supplementary material.

## Supplementary material

**Table S1**

Content of CCAP in different AC periods.

**Table S2**

Content of aroma substances in different AC periods.

**Table S3**

Correlation of microorganisms (VIP greater than 1) with CCAP and aroma substances.

**Table S4**

Representative sequences of strains A1–A4 and their corresponding ASVs.

**Figure S1**

Diversity and abundance of tobacco microorganisms. ACE and Shannon indices of bacterial **(A, B)** and fungal **(C, D)** communities. T1, fresh tobacco leaves; T2, wilting; T3, yellowing; T4, browning; T5, stem drying; T6, end of AC. ^*^, ^**^, and ^***^ denote significant differences at *P* < 0.05, *P* < 0.01 and *P* < 0.001, respectively.

**Figure S2**

PLS-DA plots of bacterial (Top 20), fungal (Top 20), CCAP, and aroma substances where R2X (cum) = 0.569, R2 (cum) = 0.808, and Q2 (cum) = 0.437 **(a)** and permutation testing of the OPLS-DA **(b)**.

**Figure S3**

Molecular identification of target microorganisms.
