# Correction: The role of microorganisms in the conversion of color class aroma precursors to aroma during air-curing of cigar tobacco leaves

**DOI:** 10.3389/fmicb.2026.1988207

**Published:** 2026-09-17

**Authors:** Songchao Zhao, Yuanyuan Li, Hongyang Si, Zhishun Chai, Yifan Li, Yunkang Lei, Junchun Gao, Mingqin Zhao, Fang Liu

**Affiliations:** 1 College of Tobacco Science, Flavors and Fragrance Engineering & Technology Research Center of Henan Province, Henan Agricultural University, Zhengzhou, China; 2 Staff Development Institute of China National Tobacco Corporation, Zhengzhou, China; 3 Cigar Fermentation Technology Key Laboratory of Tobacco Industry, Chengdu, China; 4 Danzhou City Branch of Hainan Province Tobacco Company, Danzhou, China; 5 Deyang City Branch of Sichuan Province Tobacco Company, Deyang, China; 6 Hongyun Honghe Tobacco (Group) Co., Ltd., Kunming, China

**Keywords:** air-curing, aroma components, cigar tobacco leaf, color class aroma precursors, key functional microorganisms, verification test

Affiliation China National Tobacco Corporation and Hongyun Honghe Tobacco (Group) Co., Ltd. was erroneously omitted for author SZ and JG. The **Conflict of interest** statement has been corrected to

**Conflict of interest**: SZ was employed by the Staff Development Institute of China National Tobacco Corporation. YiL was employed by Danzhou City Branch of Hainan Province Tobacco Company, YunL was employed by Deyang City Branch of Sichuan Province Tobacco Company, and JG was employed by Hongyun Honghe Tobacco (Group) Co., Ltd.

The remaining author(s) declared that this work was conducted in the absence of any commercial or financial relationships that could be construed as a potential conflict of interest.

2nd Affiliation

Staff Development Institute of China National Tobacco Corporation, Zhengzhou, China was erroneously given as College of Staff Education, China Tobacco Corporation, Zhengzhou, China

6th Affiliation

Hongyun Honghe Tobacco (Group) Co., Ltd., Kunming, China was erroneously given as Honghe City Branch of Yunnan Province Tobacco Company, Kunming, Yunnan, China

The original version of this article has been updated.

