# Supplementary material for: The role of microorganisms in the conversion of color class aroma precursors to aroma during air-curing of cigar tobacco leaves
Source: Front Microbiol. 2026 Jul 14;17:1823617. doi: 10.3389/fmicb.2026.1823617 (PMC13407536; doi:10.3389/fmicb.2026.1823617)
Supplement: Table S1 — Content of CCAP in different AC periods. [file Data_Sheet_1.docx]

Table S1 Content of CCAP in different AC periods

| Substance Category | CCAP | T1 | T2 | T3 | T4 | T5 | T6 |
| --- | --- | --- | --- | --- | --- | --- | --- |
| Chlorophyll | Chlorophyll a | 1.948±0.146 ^a^ | 1.715±0.091 ^b^ | 0.483±0.043 ^c^ | 0.191±0.044 ^d^ | 0.144±0.037 ^d^ | 0.104±0.012 ^d^ |
|  | Chlorophyll b | 0.875±0.02 ^a^ | 0.764±0.032 ^b^ | 0.165±0.017 ^c^ | 0.073±0.01 ^d^ | 0.054±0.006 ^de^ | 0.031±0.006 ^e^ |
| Carotenoids | Lutein | 0.259±0.025 ^a^ | 0.243±0.015 ^a^ | 0.189±0.01 ^b^ | 0.132±0.01 ^c^ | 0.103±0.009 ^d^ | 0.082±0.007 ^d^ |
|  | β-Carotene | 0.897±0.067 ^a^ | 0.687±0.03 ^b^ | 0.635±0.033 ^b^ | 0.542±0.047 ^c^ | 0.379±0.05 ^d^ | 0.346±0.027 ^d^ |
| Browning reaction substances | Chlorogenic acid | 12.012±0.24 ^a^ | 12.182±0.593 ^a^ | 9.012±0.293 ^b^ | 4.064±0.439 ^c^ | 2.205±0.253 ^d^ | 1.841±0.312 ^d^ |
|  | Rutin | 0.809±0.027 ^a^ | 0.824±0.019 ^a^ | 0.742±0.025 ^b^ | 0.608±0.038 ^c^ | 0.538±0.032 ^d^ | 0.511±0.035 ^d^ |
|  | Scopoletin | 0.111±0.004 ^b^ | 0.121±0.004 ^a^ | 0.109±0.003 ^b^ | 0.086±0.006 ^c^ | 0.074±0.004 ^d^ | 0.071±0.003 ^d^ |
|  | Reducing sugar | 5.682±0.357 ^a^ | 5.243±0.187 ^b^ | 3.312±0.557 ^c^ | 2.031±0.145 ^d^ | 1.647±0.118 ^de^ | 1.454±0.091 ^e^ |
|  | Amino Acid | 31.329±3.661 ^e^ | 63.521±5.667 ^c^ | 86.363±7.993 ^b^ | 139.432±13.32 ^a^ | 61.947±2.608 ^c^ | 43.856±3.388 ^d^ |
|  | Protein | 7.445±0.393 ^a^ | 6.659±0.566 ^b^ | 4.567±0.705 ^c^ | 3.343±0.485 ^d^ | 2.687±0.212 ^de^ | 2.499±0.073 ^e^ |

Values are presented as mean ± standard deviation. A significant difference is denoted by distinct letters (P < 0.05).

Table S2 Content of aroma substances in different AC periods

| Aroma category | Aroma substance | CAS | Retention time (min) | T1 | T2 | T3 | T4 | T5 | T6 |
| --- | --- | --- | --- | --- | --- | --- | --- | --- | --- |
| Browning reaction products | Furfural | 98-01-1 | 7.11 | 5.621±0.182 ^b^ | 5.184±0.044 ^c^ | 6.1±0.124 ^a^ | 5.473±0.212 ^b^ | 5.012±0.283 ^cd^ | 4.817±0.177 ^d^ |
|  | Furfuryl alcohol | 98-00-0 | 7.99 | 2.8±0.082 ^d^ | 2.633±0.032 ^d^ | 3.114±0.413 ^c^ | 4.081±0.167 ^a^ | 3.808±0.186 ^ab^ | 3.693±0.163 ^b^ |
|  | 5-Methyl furfural | 620-02-0 | 13.43 | 1.192±0.011 ^e^ | 1.867±0.007 ^c^ | 2.412±0.061 ^a^ | 1.836±0.033 ^c^ | 1.629±0.032 ^d^ | 2.31±0.066 ^b^ |
| Carotenoid degradation products | 6-Methyl-5-hepten-2-ol | 4630-06-2 | 14.60 | 1.17±0.062 ^bc^ | 1.183±0.024 ^bc^ | 1.414±0.095 ^a^ | 1.285±0.203 ^ab^ | 1.01±0.03 ^d^ | 1.084±0.039 ^d^ |
|  | 6-Methyl-5-hepten-2-one | 110-93-0 | 14.92 | 0.175±0.035 ^c^ | 0.596±0.046 ^a^ | 0.253±0.063 ^c^ | 0.351±0.075 ^b^ | n.d. | 0.411±0.063 ^b^ |
|  | Linalool | 78-70-6 | 22.21 | 0.65±0.021 ^a^ | 0.673±0.005 ^a^ | 0.594±0.031 ^ab^ | 0.5±0.078 ^c^ | 0.623±0.06 ^a^ | 0.525±0.051 ^bc^ |
|  | Safranal | 116-26-7 | 27.76 | 0.215±0.017 ^b^ | 0.242±0.01 ^b^ | 0.318±0.044 ^a^ | 0.328±0.009 ^a^ | 0.319±0.024 ^a^ | 0.215±0.048 ^b^ |
|  | β-Cyclocitral | 432-25-7 | 28.86 | 0.536±0.173 ^b^ | 0.811±0.021 ^ab^ | 0.656±0.26 ^ab^ | 1.006±0.008 ^a^ | 0.702±0.193 ^ab^ | 0.882±0.243 ^ab^ |
|  | β-Damascenone | 35044-68-9 | 36.73 | 13.573±0.217 ^e^ | 20.902±0.084 ^c^ | 23.581±0.223 ^a^ | 20.096±0.473 ^d^ | 20.35±0.308 ^d^ | 21.492±0.391 ^b^ |
|  | β -Dihydrodamascenone | 23726-91-2 | 38.00 | 5.41±0.149 ^f^ | 9.631±0.068 ^e^ | 17.912±0.199 ^d^ | 22.664±0.171 ^a^ | 18.356±0.106 ^c^ | 22.161±0.24 ^b^ |
|  | Geranylacetone | 689-67-8 | 39.55 | 1.91±0.012 ^d^ | 2.336±0.008 ^b^ | 1.882±0.074 ^d^ | 2.395±0.05 ^b^ | 2.092±0.023 ^c^ | 3.574±0.072 ^a^ |
|  | Dihydroactinidiolide | 17092-92-1 | 42.53 | 11.811±0.394 ^b^ | 13.73±0.124 ^a^ | 7.783±0.444 ^d^ | 8.02±0.368 ^d^ | 9.841±0.819 ^c^ | 13.248±0.951 ^a^ |
|  | Tabanone 1 | 13215-88-8 | 43.70 | 3.171±0.11 ^d^ | 5.852±0.082 ^b^ | 6.499±0.345 ^a^ | 5.177±0.474 ^c^ | 6.404±0.235 ^a^ | 6.062±0.375 ^ab^ |
|  | Tabanone 2 | 13215-88-8 | 44.07 | 11.307±0.357 ^e^ | 29.908±1.538 ^a^ | 23.004±0.173 ^c^ | 21.613±0.953 ^c^ | 29.02±1.373 ^a^ | 25.024±1.147 ^b^ |
|  | Tabanone 3 | 13215-88-8 | 45.73 | 2.306±0.093 ^c^ | 4.478±0.09 ^b^ | 7.45±0.464 ^a^ | 6.991±0.164 ^a^ | 7.259±0.581 ^a^ | 7.565±0.49 ^a^ |
|  | Tabanone 4 | 13215-88-8 | 46.19 | 10.547±0.439 ^d^ | 21.022±0.208 ^c^ | 32.384±1.936 ^a^ | 27.471±1.052 ^b^ | 30.673±1.607 ^a^ | 28.242±1.686 ^b^ |
|  | 3-Hydrogen-dihydrodamascone | 35734-61-3 | 45.95 | 1.07±0.037 ^c^ | 1.765±0.093 ^b^ | 1.072±0.171 ^c^ | 2.425±0.11 ^a^ | 1.465±0.033 ^b^ | 1.541±0.347 ^b^ |
|  | Farnesyl acetone | 1117-52-8 | 56.26 | 7.507±0.321 ^c^ | 9.839±0.126 ^b^ | 10.919±0.751 ^b^ | 10.854±0.825 ^b^ | 10.541±0.824 ^b^ | 15.845±1.369 ^a^ |
| Chlorophyll degradation products | Neophytadiene | 504-96-1 | 53.61 | 256.463±9.749 ^f^ | 505.983±5.084 ^e^ | 1258±82.652 ^b^ | 1511±68.542 ^a^ | 673.786±39.195 ^d^ | 1114±74.413 ^c^ |

Note: n.d. (not detected); Tabanone 1-4 shows the four isomers of tabanone; Values are presented as mean ± standard deviation. A significant difference is denoted by distinct letters (P < 0.05).

**Table S4** Representative sequences of strains A1–A4 and their corresponding ASVs

| **Name** | **Sequence** |
| --- | --- |
| **ASV17** | GTAGTCCACGCCGTAAACGATGAGTGCTAAGTGTTAGAGGGTTTCCGCCCTTTAGTGCTGCAGCTAACGCATTAAGCACTCCGCCTGGGGAGTACGGTCGCAAGACTGAAACTCAAAGGAATTGACGGGGGCCCGCACAAGCGGTGGAGCATGTGGTTTAATTCGAAGCAACGCGAAGAACCTTACCAGGTCTTGACATCCTCTGACAACTCTAGAGATAGAGCGTTCCCCTTCGGGGGACAGAGTGACAGGTGGTGCATGGTTGTCGTCAGCTCGTGTCGTGAGATGTTGGGTTAAGTCCCGCAACGAGCGCAACCCTTGATCTTAGTTGCCAGCATTTAGTTGGGCACTCTAAGGTGACTGCCGGTGACAAACCGGA |
| **ASV57** | GTAGTCCACGCCGTAAACGATGAATGCTAGGTGTTAGGGGTTTCGATACCCTTGGTGCCGAAGTTAACACATTAAGCATTCCGCCTGGGGAGTACGGTCGCAAGACTGAAACTCAAAGGAATTGACGGGGACCCGCACAAGCAGTGGAGTATGTGGTTTAATTCGAAGCAACGCGAAGAACCTTACCAGGTCTTGACATCCCTCTGACCGGTCTAGAGATAGGCCTTTCCTTCGGGACAGAGGAGACAGGTGGTGCATGGTTGTCGTCAGCTCGTGTCGTGAGATGTTGGGTTAAGTCCCGCAACGAGCGCAACCCTTATGCTTAGTTGCCAGCAGGTCAAGCTGGGCACTCTAAGCAGACTGCCGGTGACAAACCGGA |
| **ASV501** | GTAGTCCACGCCGTAAACGATGAGTGCTAAGTGTTAGAGGGTTTCCGCCCTTTAGTGCTGAAGTTAACGCATTAAGCACTCCGCCTGGGGAGTACGGCCGCAAGGCTGAAACTCAAAGGAATTGACGGGGGCCCGCACAAGCGGTGGAGCATGTGGTTTAATTCGAAGCAACGCGAAGAACCTTACCAGGTCTTGACATCCTCTGAAAACCCTAGAGATAGGGCTTCTCCTTCGGGAGCAGAGTGACAGGTGGTGCATGGTTGTCGTCAGCTCGTGTCGTGAGATGTTGGGTTAAGTCCCGCAACGAGCGCAACCCTTGATCTTAGTTGCCATCATTAAGTTGGGCACTCTAAGGTGACTGCCGGTGACAAACCGGA |
| **A1** | TATACATGCAGTCGAGCGAACTGATTAGAAGCTTGCTTCTATGACGTTAGCGGCGGACGGGTGAGTAACACGTGGGCAACCTGCCTGTAAGACTGGGATAACTTCGGGAAACCGAAGCTAATACCGGATAGGATCTTCTCCTTCATGGGAGATGATTGAAAGATGGTTTCGGCTATCACTTACAGATGGGCCCGCGGTGCATTAGCTAGTTGGTGAGGTAACGGCTCACCAAGGCAACGATGCATAGCCGACCTGAGAGGGTGATCGGCCACACTGGGACTGAGACACGGCCCAGACTCCTACGGGAGGCAGCAGTAGGGAATCTTCCGCAATGGACGAAAGTCTGACGGAGCAACGCCGCGTGAGTGATGAAGGCTTTCGGGTCGTAAAACTCTGTTGTTAGGGAAGAACAAGTACGAGAGTAACTGCTCGTACCTTGACGGTACCTAACCAGAAAGCCACGGCTAACTACGTGCCAGCAGCCGCGGTAATACGTAGGTGGCAAGCGTTATCCGGAATTATTGGGCGTAAAGCGCGCGCAGGCGGTTTCTTAAGTCTGATGTGAAAGCCCACGGCTCAACCGTGGAGGGTCATTGGAAACTGGGGAACTTGAGTGCAGAAGAGAAAAGCGGAATTCCACGTGTAGCGGTGAAATGCGTAGAGATGTGGAGGAACACCAGTGGCGAAGGCGGCTTTTTGGTCTGTAACTGACGCTGAGGCGCGAAAGCGTGGGGAGCAAACAGGATTAGATACCCTGGTAGTCCACGCCGTAAACGATGAGTGCTAAGTGTTAGAGGGTTTCCGCCCTTTAGTGCTGCAGCTAACGCATTAAGCACTCCGCCTGGGGAGTACGGTCGCAAGACTGAAACTCAAAGGAATTGACGGGGGCCCGCACAAGCGGTGGAGCATGTGGTTTAATTCGAAGCAACGCGAAGAACCTTACCAGGTCTTGACATCCTCTGACAACTCTAGAGATAGAGCGTTCCCCTTCGGGGGACAGAGTGACAGGTGGTGCATGGTTGTCGTCAGCTCGTGTCGTGAGATGTTGGGTTAAGTCCCGCAACGAGCGCAACCCTTGATCTTAGTTGCCAGCATTTAGTTGGGCACTCTAAGGTGACTGCCGGTGACAAACCGGAGGAAGGTGGGGATGACGTCAAATCATCATGCCCCTTATGACCTGGGCTACACACGTGCTACAATGGATGGTACAAAGGGCTGCAAGACCGCGAGGTCAAGCCAATCCCATAAAACCATTCTCAGTTCGGATTGTAGGCTGCAACTCGCCTACATGAAGCTGGAATCGCTAGTAATCGCGGATCAGCATGCCGCGGTGAATACGTTCCCGGGCCTTGTACACACCGCCCGTCACACCACGAGAGTTTGTAACACCCGAAGTCGGTGGAGTAACCGTAAGGAGCTAGCCGCCTAAG |
| **A2** | CTATACATGCAGTCGAGCGAGGGTTATTTAGAAGCTTGCTTCTAAATAACCTAGCGGCGGACGGGTGAGTAACACGTAGGCAACCTGCCCACAAGACAGGGATAACTACCGGAAACGGTAGCTAATACCCGATACATCCTTTTCCTGCATGGGAGAAGGAGGAAAGACGGAGCAATCTGTCACTTGTGGATGGGCCTGCGGCGCATTAGCTAGTTGGTGGGGTAAAGGCCTACCAAGGCGACGATGCGTAGCCGACCTGAGAGGGTGATCGGCCACACTGGGACTGAGACACGGCCCAGACTCCTACGGGAGGCAGCAGTAGGGAATCTTCCGCAATGGGCGAAAGCCTGACGGAGCAACGCCGCGTGAGTGATGAAGGTTTTCGGATCGTAAAGCTCTGTTGCCAGGGAAGAACGTCTTGTAGAGTAACTGCTACAAGAGTGACGGTACCTGAGAAGAAAGCCCCGGCTAACTACGTGCCAGCAGCCGCGGTAATACGTAGGGGGCAAGCGTTGTCCGGAATTATTGGGCGTAAAGCGCGCGCAGGCGGCTCTTTAAGTCTGGTGTTTAATCCCGAGGCTCAACTTCGGGTCGCACTGGAAACTGGAGAGCTTGAGTGCAGAAGAGGAGAGTGGAATTCCACGTGTAGCGGTGAAATGCGTAGAGATGTGGAGGAACACCAGTGGCGAAGGCGACTCTCTGGGCTGTAACTGACGCTGAGGCGCGAAAGCGTGGGGAGCAAACAGGATTAGATACCCTGGTAGTCCACGCCGTAAACGATGAATGCTAGGTGTTAGGGGTTTCGATACCCTTGGTGCCGAAGTTAACACATTAAGCATTCCGCCTGGGGAGTACGGTCGCAAGACTGAAACTCAAAGGAATTGACGGGGACCCGCACAAGCAGTGGAGTATGTGGTTTAATTCGAAGCAACGCGAAGAACCTTACCAGGTCTTGACATCCCTCTGACCGGTCTAGAGATAGGCCTTTCCTTCGGGACAGAGGAGACAGGTGGTGCATGGTTGTCGTCAGCTCGTGTCGTGAGATGTTGGGTTAAGTCCCGCAACGAGCGCAACCCTTATGCTTAGTTGCCAGCAGGTCAAGCTGGGCACTCTAAGCAGACTGCCGGTGACAAACCGGAGGAAGGTGGGGATGACGTCAAATCATCATGCCCCTTATGACCTGGGCTACACACGTACTACAATGGCCGGTACAACGGGAAGCGAAGCCGCGAGGTGGAGCCAATCCTAGAAAAGCCGGTCTCAGTTCGGATTGTAGGCTGCAACTCGCCTACATGAAGTCGGAATTGCTAGTAATCGCGGATCAGCATGCCGCGGTGAATACGTTCCCGGGTCTTGTACACACCGCCCGTCACACCACGAGAGTTTACAACACCCGAAGTCGGTGAGGTAACCGCAAGGAGCCAGCCGCC |
| **A3** | CTATACATGCAGTCGAGCGAATGGATTAAGAGCTTGCTCTTATGAAGTTAGCGGCGGACGGGTGAGTAACACGTGGGTAACCTGCCCATAAGACTGGGATAACTCCGGGAAACCGGGGCTAATACCGGATAACATTTTGAACCGCATGGTTCGAAATTGAAAGGCGGCTTCGGCTGTCACTTATGGATGGACCCGCGTCGCATTAGCTAGTTGGTGAGGTAACGGCTCACCAAGGCAACGATGCGTAGCCGACCTGAGAGGGTGATCGGCCACACTGGGACTGAGACACGGCCCAGACTCCTACGGGAGGCAGCAGTAGGGAATCTTCCGCAATGGACGAAAGTCTGACGGAGCAACGCCGCGTGAGTGATGAAGGCTTTCGGGTCGTAAAACTCTGTTGTTAGGGAAGAACAAGTGCTAGTTGAATAAGCTGGCACCTTGACGGTACCTAACCAGAAAGCCACGGCTAACTACGTGCCAGCAGCCGCGGTAATACGTAGGTGGCAAGCGTTATCCGGAATTATTGGGCGTAAAGCGCGCGCAGGTGGTTTCTTAAGTCTGATGTGAAAGCCCACGGCTCAACCGTGGAGGGTCATTGGAAACTGGGAGACTTGAGTGCAGAAGAGGAAAGTGGAATTCCATGTGTAGCGGTGAAATGCGTAGAGATATGGAGGAACACCAGTGGCGAAGGCGACTTTCTGGTCTGTAACTGACACTGAGGCGCGAAAGCGTGGGGAGCAAACAGGATTAGATACCCTGGTAGTCCACGCCGTAAACGATGAGTGCTAAGTGTTAGAGGGTTTCCGCCCTTTAGTGCTGAAGTTAACGCATTAAGCACTCCGCCTGGGGAGTACGGCCGCAAGGCTGAAACTCAAAGGAATTGACGGGGGCCCGCACAAGCGGTGGAGCATGTGGTTTAATTCGAAGCAACGCGAAGAACCTTACCAGGTCTTGACATCCTCTGAAAACCCTAGAGATAGGGCTTCTCCTTCGGGAGCAGAGTGACAGGTGGTGCATGGTTGTCGTCAGCTCGTGTCGTGAGATGTTGGGTTAAGTCCCGCAACGAGCGCAACCCTTGATCTTAGTTGCCATCATTAAGTTGGGCACTCTAAGGTGACTGCCGGTGACAAACCGGAGGAAGGTGGGGATGACGTCAAATCATCATGCCCCTTATGACCTGGGCTACACACGTGCTACAATGGACGGTACAAAGAGCTGCAAGACCGCGAGGTGGAGCTAATCTCATAAAACCGTTCTCAGTTCGGATTGTAGGCTGCAACTCGCCTACATGAAGCTGGAATCGCTAGTAATCGCGGATCAGCATGCCGCGGTGAATACGTTCCCGGGCCTTGTACACACCGCCCGTCACACCACGAGAGTTTGTAACACCCGAAGTCGGTGGGGTAACCTTTTTGGAGCCAGCCGCCTAAG |
| **A4** | CTATACATGCAGTCGAGCGATGGATTAAGAGCTTGCTCTTATGAAGTTAGCGGCGGACGGGTGAGTAACACGTGGGTAACCTGCCCATAAGACTGGGATAACTCCGGGAAACCGGGGCTAATACCGGATAACATTTTGAACCGCATGGTTCGAAATTGAAAGGCGGCTTCGGCTGTCACTTATGGATGGACCCGCGTCGCATTAGCTAGTTGGTGAGGTAACGGCTCACCAAGGCAACGATGCGTAGCCGACCTGAGAGGGTGATCGGCCACACTGGGACTGAGACACGGCCCAGACTCCTACGGGAGGCAGCAGTAGGGAATCTTCCGCAATGGACGAAAGTCTGACGGAGCAACGCCGCGTGAGTGATGAAGGCTTTCGGGTCGTAAAACTCTGTTGTTAGGGAAGAACAAGTGCTAGTTGAATAAGCTGGCACCTTGACGGTACCTAACCAGAAAGCCACGGCTAACTACGTGCCAGCAGCCGCGGTAATACGTAGGTGGCAAGCGTTATCCGGAATTATTGGGCGTAAAGCGCGCGCAGGTGGTTTCTTAAGTCTGATGTGAAAGCCCACGGCTCAACCGTGGAGGGTCATTGGAAACTGGGAGACTTGAGTGCAGAAGAGGAAAGTGGAATTCCATGTGTAGCGGTGAAATGCGTAGAGATATGGAGGAACACCAGTGGCGAAGGCGACTTTCTGGTCTGTAACTGACACTGAGGCGCGAAAGCGTGGGGAGCAAACAGGATTAGATACCCTGGTAGTCCACGCCGTAAACGATGAGTGCTAAGTGTTAGAGGGTTTCCGCCCTTTAGTGCTGAAGTTAACGCATTAAGCACTCCGCCTGGGGAGTACGGCCGCAAGGCTGAAACTCAAAGGAATTGACGGGGGCCCGCACAAGCGGTGGAGCATGTGGTTTAATTCGAAGCAACGCGAAGAACCTTACCAGGTCTTGACATCCTCTGAAAACCCTAGAGATAGGGCTTCTCCTTCGGGAGCAGAGTGACAGGTGGTGCATGGTTGTCGTCAGCTCGTGTCGTGAGATGTTGGGTTAAGTCCCGCAACGAGCGCAACCCTTGATCTTAGTTGCCATCATTAAGTTGGGCACTCTAAGGTGACTGCCGGTGACAAACCGGAGGAAGGTGGGGATGACGTCAAATCATCATGCCCCTTATGACCTGGGCTACACACGTGCTACAATGGACGGTACAAAGAGCTGCAAGACCGCGAGGTGGAGCTAATCTCATAAAACCGTTCTCAGTTCGGATTGTAGGCTGCAACTCGCCTACATGAAGCTGGAATCGCTAGTAATCGCGGATCAGCATGCCGCGGTGAATACGTTCCCGGGCCTTGTACACACCGCCCGTCACACCACGAGAGTTTGTAACACCCGAAGTCGGTGGGGTAACCTTTTTGGAGCCAGCCGCCTAA |


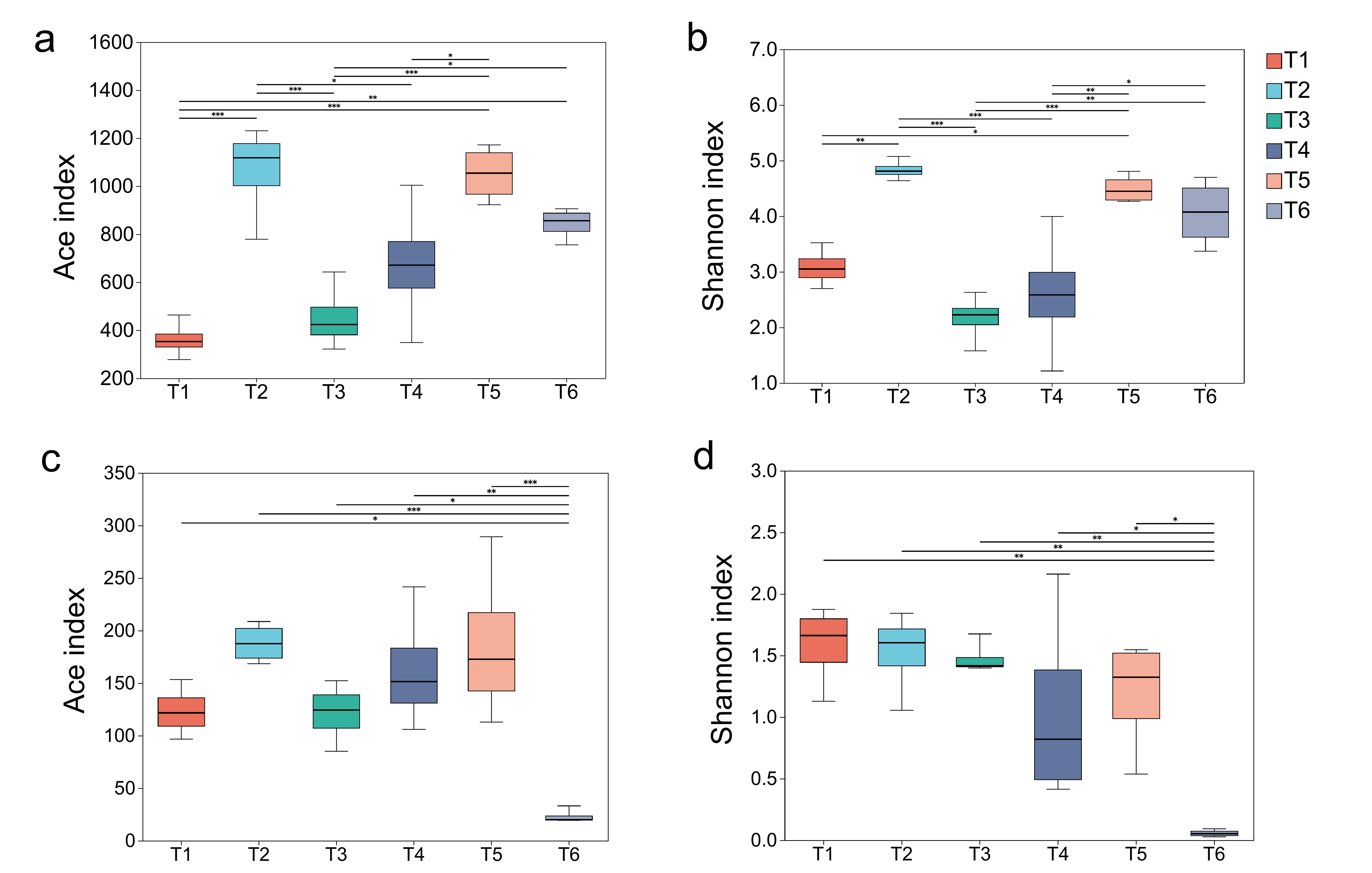


Figure S1. Diversity and abundance of tobacco microorganisms. ACE and Shannon indices of bacterial (a, b) and fungal (c, d) communities. T1, fresh tobacco leaves; T2, wilting; T3, yellowing; T4, browning; T5, stem drying; T6, end of AC. *, **, and *** denote significant differences at P < 0.05, P < 0.01 and P < 0.001, respectively.


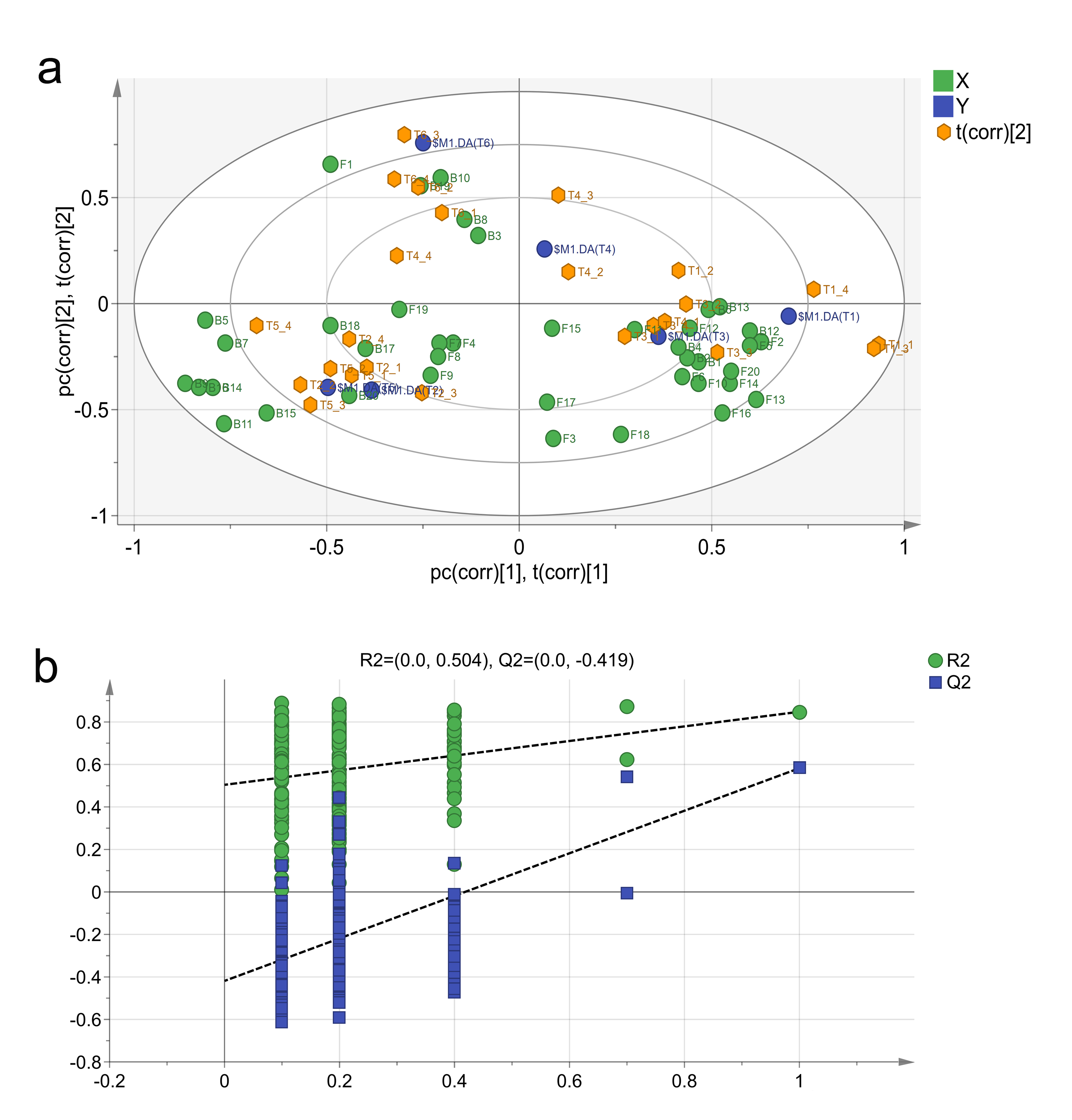


Fig. S2. PLS-DA plots of bacterial (Top 20), fungal (Top 20), CCAP, and aroma substances where R2X (cum) = 0.569, R2 (cum) = 0.808, and Q2 (cum) = 0.437 (a) and permutation testing of the OPLS-DA (b). (B1) *Pandoraea*. (B2) unclassified *Burkholderiaceae*. (B3) *Pseudomonas*. (B4) *Rhodococcus*. (B5) *Sphingomonas*. (B6) unclassified *Comamonadaceae*. (B7) *Massilia*. (B8) *Pantoea*. (B9) *Bacillus*. (B10) *Limnobacter*. (B11) *Nocardioides*. (B12) *Cloacibacterium*. (B13) *Cupriavidus*. (B14) unclassified *Planococcaceae*. (B15) *Exiguobacterium*. (B16) *Blastococcus*. (B17) *Lactobacillus*. (B18) unclassified *Burkholderiales*. (B19) norank *Gaiellales*. (B20) *Paenibacillus*. (F1) *Alternaria*. (F2) *Filobasidium*. (F3) *Cladosporium*. (F4) *Aspergillus*. (F5) *Symmetrospora*. (F6) *Curvularia*. (F7) *Colletotrichum*. (F8) unclassified *Didymellaceae*. (F9) *Cercospora*. (F10) *Erythrobasidium*. (F11) *Pseudopithomyces*. (F12) *Sporidiobolus*. (F13) *Coprinellus*. (F14) *Apiotrichum*. (F15) *Epicoccum*. (F16) *Meyerozyma*. (F17) *Cystobasidium*. (F18) unclassified Fungi. (F19) *Stemphylium*. (F20) *Hormographiella*.


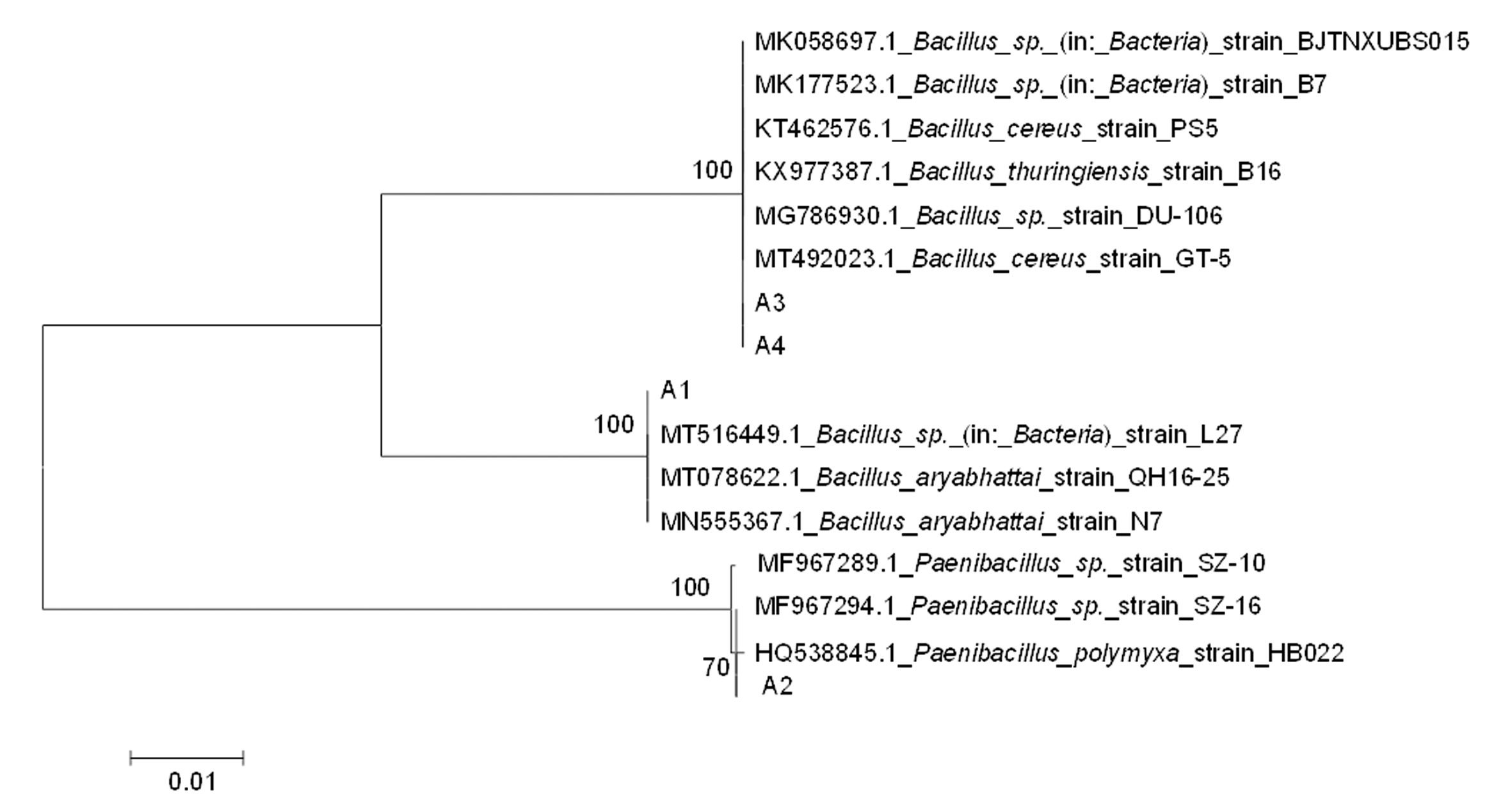


Fig. S3. Molecular identification of target microorganisms.
